# Relationships of leisure-time and non-leisure-time physical activity with depressive symptoms: a population-based study of Taiwanese older adults

**DOI:** 10.1186/1479-5868-9-28

**Published:** 2012-03-14

**Authors:** Li-Jung Chen, Clare Stevinson, Po-Wen Ku, Yu-Kai Chang, Da-Chen Chu

**Affiliations:** 1 Department of Exercise Health Science, National Taiwan University of Physical Education and Sport, No. 16, Section 1, Shuang-Shih Rd, Taichung 404, Taiwan; 2 School of Sport, Exercise and Health Sciences, Loughborough University, Leicestershire LE11 3TU, UK; 3 Graduate Institute of Sports and Health, National Changhua University of Education, No. 1 Jin-De Rd, Changhua 500, Taiwan; 4 Institute of Public Health, National Yang-Ming University, No.155, Sec.2, Linong Street, Taipei 112, Taiwan; 5 Graduate Institute of Coaching Science, National Taiwan Sport University, No.250, Wenhua 1st Rd., Guishan Shiang, Taoyuan County 333, Taiwan; 6 Department of Neurosurgery, Taipei City Hospital, No.145, Zhengzhou Rd., Datong District, Taipei 103, Taiwan

**Keywords:** Exercise, Depression, Aging, Intensity, Non leisure-time physical activity

## Abstract

**Background:**

Limited research has explored the relationship between non-leisure-time physical activity (NLTPA), including domestic and work-related physical activities, with depressive symptoms. This study was designed to elucidate independent associations between leisure-time physical activity (LTPA), NLTPA, and specific parameters of physical activity (frequency, duration and intensity) with depressive symptoms in older adults.

**Methods:**

A total of 2,727 persons aged ≥ 65 years participating in the 2005 Taiwan National Health Interview Survey were studied. Depressive symptoms were measured by the Center for Epidemiological Studies Depression Scale. Information regarding energy parameters for each type of LTPA and NLTPA during the past 2-week period was analyzed. After adjusting for socio-demographic variables, lifestyle behaviors and health status, multivariate logistic regression models were used to compute adjusted odds ratios (AOR) for LTPA and NLTPA for predicting depressive symptoms.

**Results:**

LTPA but not NLTPA was significantly associated with depressive symptoms. Compared with participants expending 2000+ kcal/week through LTPA, the risk of experiencing depressive symptoms was significantly higher for those expending 1-999 kcal/week (AOR = 2.06, 95% CI: 1.25-3.39), and those who expending 0 kcal/week (AOR = 3.72, 95%CI: 2.28-6.06). Among the three parameters of LTPA (intensity, duration and frequency) examined, only intensity was independently associated with depressive symptoms.

**Conclusions:**

These findings imply that exercise recommendations for older adults should emphasize the importance of higher intensity activity, rather than frequency or duration, for improved mental well-being. However, well-designed prospective cohort studies or intervention trials are needed to confirm these findings.

## Background

Depression is a common mental disorder, and projected to become the single highest contributor to the global disease burden by 2030, above heart disease, stroke and HIV/AIDS [1]. Depressive symptoms are particularly prevalent in later life, and are associated with decreased physical, cognitive and social functioning, and overall greater morbidity and mortality [2]. Consequences include loss of independence, personal and family suffering, and increasing societal burden in terms of public expenditure on health and social care [3]. Identifying factors that contribute to preventing depression or alleviating symptoms in this population would have important implications for public health [4].

Physical activity is one modifiable factor for which there is a growing evidence base for both protective and therapeutic effects across a range of physical and mental illnesses, with these benefits equally available for older people [5-8]. The relationship between depression and physical activity among older adults has been widely researched with results generally indicting an inverse association in observational studies [9-11], and a reduction in symptoms in randomized trials [12]. However, physical activity in most of these studies has usually denoted leisure-time physical activity (LTPA). Less attention has been paid to other aspects of lifestyle physical activity, such as domestic, commuting and occupational physical activity [9], although the potential health-enhancing role of physical activity in these contexts is increasingly recognized [13-15]. Furthermore, these types of activity generally form part of daily routines for older adults, reflecting independence, good physical function, and engagement with social networks.

Several studies have suggested that a single type of non leisure-time physical activity (NLTPA), such as walking, is related to a lower risk of depressive symptoms in older populations [16,17], and in women aged 18-45 [18]. In contrast, other studies have found that greater duration of work-related physical activity [19], and more occasions of vigorous domestic physical activity [20], are associated with a higher risk of depression/depressive symptoms. While these studies were not specific to older adults, it seems that overall there is a variable relationship between different physical activity types and depressive disorders. Moreover, other research has recorded overall physical activity levels across different activities, often in terms of energy expenditure or composite scores over a specified period [21,22]. These make it difficult to disentangle the independent associations of physical activities incurred in different contexts with the risk of depressive symptoms. Additionally, most studies have provided simple comparisons between no or low physical activity and high physical activity. Few have investigated the independent associations of frequency, intensity or duration of physical activity with depressive symptoms. Each of these components may offer diverse mechanisms by which activity may affect depressive symptoms [23].

To address some of these shortfalls, the purposes of the current study were to: (a) assess the relationships among LTPA, NLTPA and depressive symptoms with a nationally representative sample of Taiwanese older adults; (b) examine the independent associations of frequency, intensity and duration with depressive symptoms.

## Methods

### Data and sample

The analysis was based on cross-sectional data from the 2005 Taiwan National Health Interview Survey conducted by the National Health Research Institutes and Bureau of Health Promotion, Taiwan. This involved a multistage stratified systematic sampling design with probability proportional to size (PPS), based on the degree of urbanization, geographic location and administrative boundaries, to select a nationally representative sample. The target population for the survey was the 22,615,307 persons whose households were registered in any one of the 23 counties or cities in Taiwan in the end of 2004. Among the 358 townships, villages or districts from 23 counties or cities of Taiwan, a total of 187 were sampled, representing 30,680 persons. Respondents from three age groups (< 12, 12-64, 65+ years) were interviewed in their homes, providing a total sample of 24,726 participants (80.59% response rate) [24]. Only participants aged 65 and older were included in the current analysis (n = 2,724, male: 1348).

### Measures

#### Outcome variable

Depressive symptoms were measured using a 10-item Chinese version of the original 20-item CES-D (Center for Epidemiologic Studies Depression Scale) [25]. The sum of the 10 items has a potential range of 0 to 30. Participants scoring 10 or above were categorized as having depressive symptoms [11]. The 10-item Chinese version of the CES-D is consistent with the modified 11-item version of the CES-D (except one item: I felt that people dislike me), which is highly correlated with the standard 20-item version (r = 0.95) [26]. The short Chinese CES-D has also shown to function well in older Taiwanese populations [27]. Cronbach's alpha reliability coefficients for the 10-item CES-D in the present survey was 0.85, which is comparable with a 10-item Chinese version developed in Hong Kong older adults (0.78-0.79) [28].

#### Exposure variable

Two types of physical activity were self-reported. LTPA was assessed using the following question. 'Have you taken part in any sport or exercise activities in the past 2 weeks?' Respondents were asked to identify the types of physical activity they had engaged in from 31 named activities (e.g. walking, jogging, swimming), and were able to specify up to five types. They also provided frequency and duration for each activity. Metabolic equivalent (MET) intensity levels for each activity were assigned [29,30]. Energy expenditure (kcal) of each activity per week was calculated by: activity intensity code (kcal/min) × frequency (times) × duration for each time (min)/2. The energy expenditure values were then added to provide total weekly amount of energy expenditure. Participants were classified into four groups (0, 1-999, 1000-1999, ≥ 2000 kcal/week) [31]. Participation in NLTPA was assessed using the following questions. 'Have you taken part in any work-related physical activity in the past 2 weeks?' Respondents were then asked to identify the type of physical activity they engaged in from 10 named activities (e.g. farm work, heavy lifting, fishing work, household chores) and could specify up to five types. As described above, the total weekly amount of energy expenditure for NLTPA was then estimated. The sum of weekly LTPA and NLTPA was computed to provide total weekly energy expenditure. The physical activity questions were previously utilized in others studies [27,32,33], with repeated administrations for 72 participants at 1, 3, and 6 months post-interview. Consistency of the main physical activity question was 0.88, while Kappa scores for repeated measures of reported frequency and duration ranged between 0.41 and 0.46, demonstrating acceptable reliability of the questions adopted by the study [32,33].

#### Covariates

Based on previous literature [4,11,34], potential confounders requiring adjustment were identified as: (i) socio-demographical factors: sex, age (65-74, 75-84, ≥ 85), education level attained (illiterate, literate but no formal schooling, primary, secondary, college or above), marital status (widowed, separated/divorced, never married, married/cohabiting), working status (unable to work, retired, housekeeper, currently working), monthly income (New Taiwan Dollars: 0, 1-9999, 10000-19999, ≥ 20000), living status (alone versus with families/other); (ii) lifestyle behaviors: alcohol consumption (yes versus no), smoking (daily, sometimes, quit, never smoked); and (iii) health status: body mass index (BMI) (underweight < 18.5, normal 18.5-23.99, overweight 24-26.99, obese ≥ 27) [35], number of chronic diseases (ten types comprising hypertension, diabetes, hyperlipidemia, stroke, heart disease, falls, chronic respiratory disease, chronic hepatitis, cancer and arthritis), and activities of daily living (ADL) (some or great difficulties versus no difficulties at all). These were included in the subsequent regression analyses.

### Data analyses

Descriptive statistics were used to characterize the participants and cross-tabulate the relationships between depressive symptoms and different types of physical activity, and other variables. Chi-square tests were used to identify the underlying correlates. Adjusted odds ratios (AORs) were calculated for the association between overall physical activity and depressive symptoms (1 = yes, 0 = no), after controlling for potential confounders. The variables showing significant associations with depressive symptoms in Chi-square tests were entered into the multivariate logistic regression models simultaneously. The same procedures for assessing the independent effect of LTPA and NLTPA were then performed. Finally, given the significant association for LTPA and depressive symptoms, the relative contributions of activity frequency, intensity, and duration at leisure were examined in multivariate logistic regression models. Given the inability to determine and estimate their overall activity frequency, intensity and duration during leisure time, participants engaging in more than one type of LTPA (n = 217) were excluded in the three-component analysis. The three parameters were all computed in units of approximately one standard deviation difference in the models for comparability. With multivariate adjustment, separate models for the three variables were examined to assess their independent associations with depressive symptoms.

Sensitivity analyses were performed to examine the influence of missing data, and Little's MCAR (missing completely at random) test indicated that the missing pattern was not random (*p *< 0.05). The missing values of the exposure variable, the outcome variable and all covariates were estimated and replaced using the EM (Expectation Maximization) procedure based on Maximum Likelihood estimation [36]. After that, the preceding multivariate logistic regression models for estimating AOR were all repeated. The interactions between LTPA, NLTPA and covariates were assessed, but not found to be significant (*p >*0.05). Multi-collinearity checks confirmed that this was not a problem for these analyses [37,38]. All analyses were conducted using SPSS 16.0.

## Results

The overall prevalence of depressive symptoms was 20.6% in this sample of Taiwanese older adults (aged ≥ 65 years). The cross-tabulation of depressive symptoms with the underlying covariates is shown in Table 1. Apart from age (*p *= 0.768) and smoking (*p *= 0.124), all variables were significantly associated with depressive symptoms (*p *< 0.05). Participants with depressive symptoms tended to be female, separated or divorced, unable to work, living alone, and have lower educational attainments and income. Furthermore, they were more likely to be non-drinkers, underweight, have at least 3 chronic diseases, have some or great difficulties in activities of daily living, and report low levels of total, leisure-time, and non-leisure-time physical activity.

**Table 1:** Characteristics of participants aged 65 or older with depressive symptoms in Taiwan

Variables	n	Depressive Symptoms(%)	χ^2 ^test
***Socio-demographic***			

**Sex**			*p *< 0.001

Female	1225	24.5	

Male	1236	16.7	

**Age**			*p *= 0.768^a^

85+	105	21.0	

75-84	821	20.1	

65-74	1535	20.8	

**Education level**			*p *< 0.001

Illiterate	828	28.7	

Literate but no formal schooling	128	16.4	

Primary school	986	18.5	

Secondary school	352	14.2	

College+	166	9.0	

**Marital status**			*p *< 0.001

Widowed	740	25.3	

Separated/divorced	147	29.3	

Never married	68	25.0	

Married/cohabiting	1502	17.2	

**Working status**			*p *< 0.001

Unable to work	70	30.0	

Retired	1531	21.4	

Housekeeper	467	22.9	

Currently working	392	13.3	

**Monthly income (NT dollar)**			*p *< 0.001^a^

0	310	20.0	

1-9999	1406	25.0	

10000-19999	381	16.0	

20000+	346	7.8	

**Living status**			*p *< 0.001

Alone	250	31.2	

With family/others	2211	19.4	

***Lifestyle behaviors***			

**Leisure-time physical activity**			*p *< 0.001^a^

0 (kcal/wk)	1046	29.0	

1-999	743	18.6	

1000-1999	366	11.5	

2000+	304	7.9	

**Non Leisure-time physical activity**			*p *< 0.001^a^

0 (kcal/wk)	1973	22.3	

1-999	186	14.5	

1000-1999	68	11.8	

2000+	231	14.3	

**Overall physical activity**			*p *< 0.001^a^

0 (kcal/wk)	792	32.4	

1-999	739	20.0	

1000-1999	384	11.5	

2000+	546	10.6	

**Drinking**			*p *< 0.001

Yes	503	13.9	

No	1958	22.3	

**Smoking**			*p *= 0.124

Current smoker	438	20.4	

Quit	247	15.8	

Never smoked	1775	21.4	

***Health Status***			

**Body mass index**			*p *< 0.001

Underweight < 18.5	155	33.5	

Obese 27+	474	20.5	

Overweight 24-26.99	677	17.9	

Normal 18.5-23.99	1120	20.4	

**Number of chronic diseases**			*p *< 0.001

3+	578	31.1	

2	537	19.9	

1	690	18.4	

0	656	14.2	

**Activities of daily living**			*p *< 0.001

Some or great difficulties	234	47.9	

No difficulties at all	2227	17.7	

a: test for linear trend

Case numbers do not always total 2727 due to missing values for some variables.

Compared with those who expended energy of at least 2000 kcal/week in overall physical activity, sedentary participants tended to have higher risk of depressive symptoms (0 kcal/wk: AOR = 2.98, 95%CI: 2.02-4.41; 1-999 kcal/wk: AOR = 1.76, 95%CI: 1.18-2.63 in the fully adjusted model based on original data). These results were replicated in the fully adjusted model after replacing missing values (See Table 2).

**Table 2:** Multivariate logistic regression for AORs of overall physical activity for predicting depressive symptoms

Variables	Original Data			Replacing Missing Values		
	**n**	**AOR^a^**	**95% CI**	**n**	**AOR**	**95% CI**

***Socio-demographic***						

**Sex**			*p *= 0.204			*p *= 0.604

Female	1190	1.20	0.90-1.60	1378	1.07	0.83-1.39

Male	1212	1		1349	1	

**Education level**			*p *= 0.056			*p *= 0.004

Illiterate	799	1.80	0.92-3.50	973	2.30*	1.22-4.34

Literate but no formal schooling	125	1.12	0.50-2.52	156	1.23	0.58-2.57

Primary school	968	1.29	0.68-2.47	1041	1.66	0.90-3.09

Secondary school	348	1.14	0.57-2.26	381	1.46	0.76-2.80

College+	162	1		176	1	

**Marital status**			*p *= 0.016			*p *= 0.002

Widowed	718	0.86	0.66-1.13	886	0.91	0.71-1.17

Separated/divorced	146	1.84**	1.17-2.90	167	2.01**	1.34-3.01

Never married	67	0.97	0.50-1.90	77	1.03	0.56-1.89

Married/cohabiting	1471	1		1597	1	

**Working status**			*p *= 0.345			*p *= 0.390

Unable to work	68	1.32	0.64-2.70	91	1.12	0.62-2.03

Retired	1502	1.16	0.79-1.72	1689	1.08	0.76-1.53

Housekeeper	454	0.90	0.56-1.43	549	0.85	0.56-1.29

Currently working	378	1		398	1	

**Monthly income (NT dollar)**			*p *< 0.001			*p *< 0.001

0	302	2.05*	1.17-3.59	379	2.32**	1.41-3.82

1-9999	1376	2.82***	1.73-4.60	1574	2.47***	1.58-3.86

10000-19999	378	1.78*	1.04-3.06	416	1.65*	1.01-2.70

20000+	346	1		358	1	

**Living status**			*p *< 0.001			*p *< 0.001

Alone	236	2.23***	1.52-3.26	276	2.03***	1.45-2.85

With family/others	2166	1		2451	1	

***Lifestyle behaviors***						

**Overall physical activity**			*p *< 0.001			*p *< 0.001

0 (kcal/wk)	766	2.98***	2.02-4.41	1003	2.64***	1.89-3.69

1-999	723	1.76**	1.18-2.63	777	1.58*	1.11-2.23

1000-1999	379	0.94	0.58-1.55	392	0.99	0.64-1.52

2000+	534	1		555	1	

**Drinking**			*p *= 0.162			*p *= 0.353

Yes	497	0.79	0.56-1.10	526	0.87	0.64-1.17

No	1905	1		2201	1	

***Health Status***						

**Body mass index**			*p *= 0.075			*p *= 0.066

Underweight < 18.5	152	1.45*	0.95-2.21	194	1.27	0.90-1.81

Obese 27+	471	0.81	0.60-1.10	441	0.82	0.63-1.09

Overweight 24-26.99	672	0.87	0.66-1.14	1054	0.82	0.64-1.05

Normal 18.5-23.99	1107	1		1038	1	

**Number of chronic diseases**			*p *< 0.001			*p *< 0.001

3+	567	2.79***	2.00-3.88	690	3.15***	2.34-4.24

2	528	1.62**	1.14-2.29	585	1.69**	1.23-2.32

1	672	1.32	0.94-1.84	754	1.26	0.93-1.71

0	635	1		698	1	

**Activities of daily living**			*p *< 0.001			*p *< 0.001

Some or great difficulties	225	2.54***	1.84-3.51	400	2.67***	2.05-3.48

No difficulties at all	2177	1		2327	1	

a: Adjusted Odds Ratio; ***: *p *< .001; **: *p *< .01; *: *p *< .05

Case numbers do not always total 2727 due to missing values for some variables

Omnibus model *χ*^2 ^test (*p *< 0.001) and Hosmer & Lemeshow test (*p *> 0.05) were checked

In Table 3, both LTPA and NLTPA were included in the fully adjusted model based on original data first. LTPA was significantly associated with depressive symptoms in both models. With multivariate adjustment, older adults who reported no LTPA had a higher risk of depressive symptoms (0 kcal/week: AOR = 3.72, 95%CI: 2.28-6.06; 1-999 kcal/week: AOR = 2.06, 95%CI: 1.25-3.39, reference: 2000 + kcal/week). In contrast, NLTPA was not found to be significant in the fully adjusted model. Similar results were observed in the model based on missing value imputation.

**Table 3:** Multivariate logistic regression for AORs of LTPA and NLTPA for predicting depressive symptoms

Variables	Original Data			Replacing Missing Values		
	**n**	**AOR^a^**	**95% CI**	**n**	**AOR**	**95% CI**

***Socio-demographic***						

**Sex**			*p *= 0.197			*p *= 0.580

Female	1188	1.21	0.91-1.61	1378	1.08	0.83-1.40

Male	1209	1		1349	1	

**Education level**			*p *= 0.083			*p *= 0.009

Illiterate	798	1.70	0.87-3.33	973	2.17*	1.14-4.11

Literate but no formal schooling	125	1.09	0.49-2.45	156	1.17	0.56-2.48

Primary school	965	1.24	0.65-2.38	1041	1.59	0.85-2.96

Secondary school	347	1.09	0.55-2.19	381	1.42	0.73-2.73

College+	162	1		176	1	

**Marital status**			*p *= 0.011			*p *= 0.001

Widowed	717	0.868	0.66-1.14	886	0.93	0.72-1.19

Separated/divorced	146	1.92**	1.21-3.02	167	2.13***	1.41-3.20

Never married	67	0.98	0.50-1.92	77	1.07	0.58-1.97

Married/cohabiting	1467	1		1597	1	

**Working status**			*p *= 0.212			*p *= 0.225

Unable to work	68	1.51	0.72-3.15	91	1.29	0.70-2.36

Retired	1500	1.37	0.90-2.08	1689	1.32	0.90-1.92

Housekeeper	453	1.05	0.65-1.72	549	1.04	0.67-1.61

Currently working	376	1		398	1	

**Monthly income (NT dollar)**			*p *< 0.001			*p *= 0.001

0	301	2.00*	1.14-3.51	379	2.26**	1.37-3.73

1-9999	1374	2.74***	1.68-4.48	1574	2.39***	1.52-3.74

10000-19999	376	1.82*	1.06-3.13	416	1.66*	1.01-2.72

20000+	346	1		358	1	

**Living status**			*p *< 0.001			*p *< 0.001

Alone	235	2.26***	1.54-3.31	276	2.08***	1.48-2.92

With family/others	2162	1		2451	1	

***Lifestyle behaviors***						

**Leisure-time physical activity**			*p *< 0.001			*p *< 0.001

0 (kcal/wk)	1012	3.72***	2.28-6.06	1264	3.61***	2.27-5.73

1-999	727	2.06**	1.25-3.39	782	2.02**	1.25-3.25

1000-1999	361	1.24	0.70-2.20	372	1.37	0.79-2.36

2000+	297	1		309	1	

**Non-leisure-time physical activity**			*p *= 0.182			*p *= 0.342

0 (kcal/wk)	1919	1.31	0.73-2.38	2229	1.32	0.84-2.07

1-999	185	0.99	0.45-2.17	193	1.03	0.57-1.89

1000-1999	67	0.34	0.07-1.57	72	0.84	0.35-1.99

2000+	226	1		233	1	

**Drinking**			*p *= 0.155			*p *= 0.367

Yes	495	0.78	0.56-1.10	526	0.87	0.65-1.18

No	1902	1		2201	1	

***Health Status***						

**Body Mass Index**			*p *= 0.106			*p *= 0.104

Underweight < 18.5	152	1.43	0.94-2.18	194	1.24	0.87-1.76

Obese 27+	469	0.82	0.61-1.12	441	0.83	0.63-1.10

Overweight 24-26.99	671	0.88	0.67-1.16	1054	0.83	0.64-1.06

Normal 18.5-23.99	1105	1		1038	1	

**Number of Chronic diseases**			*p *< 0.001			*p *< 0.001

3+	564	2.80***	2.01-3.90	690	3.21***	2.38-4.32

2	527	1.63**	1.15-2.31	585	1.74**	1.27-2.40

1	672	1.33	0.95-1.86	754	1.27	0.94-1.73

0	634	1		698	1	

**Activities of Daily Living**			*p *< 0.001			*p *< 0.001

Some or great difficulties	225	2.53***	1.83-3.49	400	2.66***	2.04-3.47

No difficulties at all	2172	1		2327	1	

a: Adjusted Odds Ratio; ***: *p *< .001; **: *p *< .01; *: *p *< .05

Case numbers do not always total 2727 due to missing values for some variables

Omnibus model *χ*^2 ^test (*p *< 0.001) and Hosmer & Lemeshow test (*p *> 0.05) were checked

The results of the relationship of three parameters of LTPA with depressive symptoms are shown in Table 4. After adjusting other potential confounders, significant associations with depressive symptoms were observed in each models for frequency (*p *< 0.001), duration (*p *< 0.001), and intensity (*p *< 0.001). However, when the three components were all included in the same model, only the association for physical activity intensity remained significant (*p *= 0.003), indicating that participants engaging in LTPA at higher intensity is associated with reduced risk of depression. This result was also supported by the sensitivity analysis based on missing value replacement.

**Table 4:** Multivariate logistic regression for AORs of three components of LTPA for predicting depressive symptoms

Components	Original Data^a^: one-component models		Original Data^b^: three-component model		Replacing Missing Values^b^: three-component model	
	AOR	95% CI	AOR	95% CI	AOR	95% CI

**Frequency**	*p *< 0.001		*p *= 0.766		*p *= 0.522	

Per 3 sessions/week^c^	0.74	0.67-0.83	0.98	0.83-1.15	0.95	0.82-1.11

**Duration**	*p *< 0.001		*p *= 0.066		*p *= 0.103	

Per 30 min/session^c^	0.69	0.61-0.79	0.86	0.73-1.01	0.88	0.75-1.03

**Intensity**	*p *< 0.001		*p *= 0.003		*p *= 0.003	

Per 2 kcal/min^c^	0.60	0.51-0.70	0.70	0.56-0.89	0.71	0.56-0.89

AOR: Adjusted Odds Ratio

a. Each model comprised only one component. These models were conducted separately with adjustment for all covariates, including sex, education, marital status, working status, income, living alone, drinking, BMI, number of chronic diseases, ADL

b. Three components and all covariates were included in the same model

c. These units are approximately equal to one standard deviation

## Discussion

This study showed that the prevalence of depressive symptoms among older Taiwanese adults in 2005 was 20.6%. It is comparable with the 20.2% reported in another study from 2003 with a nationally representative sample of 2,570 older Taiwanese adults [39]. The current study showed that physical activity performed during leisure time is associated with a lower prevalence of depressive symptoms in this national sample of Taiwanese older adults. No association was evident for NLTPA. These findings support those from a previous study, where only LTPA was related to lower depression [40] but this was based on a younger (18-64 years) female-only sample. Additionally, among the three components of LTPA, only intensity, especially at higher intensity, seems to be associated with reduced risk of depressive symptoms, which is consistent with the findings of a recent systematic review [9].

The various settings in which physical activity takes place provide different exposures, or degrees of exposure, to the range of bio-psychosocial mechanisms operating in exercise. It has been suggested that NLTPA, such as domestic, commuting and occupational activities, are more likely to be obligatory, repetitive, or routine, while LTPA may offer a sense of enjoyment, fulfillment, connection and social interaction [40]. It has been suggested that improvements in mental health after engaging in LTPA are partially related to social relations and mutual support derived from LTPA undertaken in a group or social setting [41]. Furthermore, NLTPA, such as domestic or work-related physical activity, are often performed at a shorter duration and/or insufficient intensity than that required for yielding health benefits. The current physical activity guidelines proposed by the US [5] and the UK governments [6], and the American College of Sports Medicine [42] state that older adults should engage in 150 minutes a week of moderate-intensity, or 75 minutes a week of vigorous-intensity physical activity, or an equivalent combination of moderate- and vigorous-intensity physical activity.

Few studies have focused on the association between components of energy expenditure of physical activity and depressive symptoms in older adults. Energy expenditure is the product of frequency, intensity, and duration. The inter-relations among the three components contribute to the complexity of dose-response analyses in physical activity and morbidity. Without appropriate analyses, such as adjusting the volume of physical activity energy expenditure or the other two parameters in a multivariate model, findings may be distorted [43]. Although each of the three components contribute to the determination of energy expended through physical activity, the present study showed that only intensity emerged as an independent predictor of depressive symptoms after adjusting for frequency, duration and other confounders. Participation in LTPA, particularly at higher intensity, is associated with a reduced risk of depressive symptoms regardless of the frequency or the duration of activity. This is consistent with the physical activity recommendations for older adults suggesting that physical activity needs to be moderate or vigorous intensity for health benefits [5,6,8].

Previous research has led to inconsistent results regarding physical activity intensity and depressive disorders. Some reviewers have suggested that physical activity intensity may not be an underlying factor for preventing or mitigating depressive symptoms given that mental health benefits have been observed at various levels of intensity [44,45]. Two meta-analyses indicated that lower intensity supervised physical activity intervention may be more effective in reducing depressive mood than moderate intensity interventions among the elderly [12,46]. The dual-mode model of exercise and mood [47,48] may provide some clarification of these mixed results, suggesting that both low and high intensity can produce positive affective changes from pre-exercise to post-exercise. It is worth noting these hypotheses were not specifically aimed at, or tested in, older populations. Furthermore, most observational and intervention research of physical activity intensity has examined physical activity of different durations. This makes it difficult to compare the results of different studies and to disentangle the influence of intensity from the effect of duration [9].

Interpretations of the current results need to be treated with caution given the cross-sectional research design. Although LTPA is related to a lower risk of depressive disorder, it is not possible to ascertain if low activity levels contribute to depression, or if the presence of depressive symptoms results in decreased levels of physical activity due to feelings of low energy and motivation [34]. The potential reciprocal associations should be taken into account when interpreting the results. Furthermore, it is worth noting that as well as being self-reported and thus susceptible to recall and misclassification biases, the measure of NLTPA does not include transport-related physical activity. The relationship of this form of activity with depressive symptoms cannot be determined from the current study. Well-designed prospective cohort studies, using objective measures of physical activity such as accelerometry, or extended randomized controlled trials, are needed to establish whether LTPA at higher intensity can prevent or delay depressive symptoms in older adults, and to elucidate why intensity is relatively more important than the other two components. It is also important to specifically compare physical activity interventions at various intensities by matching on total energy expenditure or on frequency and duration, to identify optimal intensity. Nonetheless, the current study provides important preliminary data on the complex issue of dose-response effects in the physical activity/depression relationship, particularly in older adults. Furthermore, it adds to the very limited evidence base for physical activity and older adults in East Asian countries and specifically with a Taiwanese population.

## Conclusions

In summary, these results suggest that LTPA (but not NLTPA), especially at higher intensity, is associated with lower risk of depressive symptoms among older adults in a population-based sample. If physical activity has a causal role in preventing or reducing depression, these findings imply that exercise recommendations should emphasize the importance of higher intensity activity, rather than frequency or duration. However, well-designed prospective cohort studies or intervention trials are needed to justify these findings due to the cross-sectional nature of the study design.

## Abbreviations

LTPA: Leisure-time physical activity; NLTPA: Non-leisure-time physical activity.

## Competing interests

The authors declare that they have no competing interests.

## Authors' contributions

LC reviewed the literature, drafted and revised the manuscript, participated in the statistical analysis and interpretation. CS assisted in the statistical analysis and critically revised the manuscript. PK performed the statistical analysis and drafted the manuscript. YC and DC assisted in the analysis and helped to draft the manuscript. All authors read and approved the final manuscript.
